# Adaptive BDS RTK Positioning with Azimuth-Integer-Based Elevation Masking for Real-Time Deformation Monitoring in Mining Environments

**DOI:** 10.3390/s26113347

**Published:** 2026-05-25

**Authors:** Lei Zhu, Ming Li, Jingang Zhao, Baoqiang Chen, Zhenhua An, Pengfei Zhang

**Affiliations:** 1 China Coal Xi’an Design Engineering Co., Ltd., Xi’an 710054, China; 2 College of Geodesy and Geomatics, Shandong University of Science and Technology, Qingdao 266590, China

**Keywords:** azimuth-integer elevation mask, BDS RTK, mining deformation monitoring, ambiguity resolution

## Abstract

Real-time kinematic (RTK) positioning in open-pit mining environments is critically compromised by non-line-of-sight (NLOS) signals and anisotropic multipath effects induced by pit walls, haul roads, and industrial infrastructure. Conventional elevation-dependent stochastic models fail to discriminate between geometrically favorable low-elevation satellites and those subject to directional obstruction, resulting in degraded ambiguity resolution and decimeter-level positioning errors that undermine safety-critical deformation monitoring. This paper presents an adaptive RTK positioning framework utilizing azimuth-integer-based elevation masking to explicitly model site-specific obstruction geometry. The proposed method discretizes the horizontal plane into 360 integer-degree sectors, extracts minimum elevation angles per sector from 24 h line-of-sight (LOS) data, and constructs a smoothed 360°mask profile via moving-window filtering. A virtual elevation-angle transformation is introduced to normalize satellite geometry relative to the local mask, enabling adaptive down-weighting of diffraction-susceptible observations within the stochastic model without requiring multi-day satellite repeat arcs or hardware modifications. The approach was validated using 54 h of BDS data collected at eight monitoring stations within the Wangjialing open-pit mine, China. Implementation of the mask model engendered a selective 8.1% reduction in satellite participation (15.66 to 14.39 satellites) while significantly enhancing observation quality. The ambiguity validation ratio improved by 19.5% (from 9.43 to 11.27 in the experimental project), and the fix success rate increased from 92.4% to 97.2% (exceeding the 95% reliability threshold at all stations). The RMS errors in the east, north, and up directions improved by 34.8% to 65.2%, 28.7% to 77.0%, and 44.8% to 70.8%, respectively, with the most dramatic gains observed at stations subject to severe azimuthal obstruction (e.g., ZDH6 vertical RMS: from 50.7 mm to 14.8 mm). By explicitly modeling anisotropic obstruction geometry through discrete angular sampling, the proposed method achieves sub-centimeter positioning accuracy and robust ambiguity resolution in challenging mining environments without additional hardware or empirical threshold tuning, offering a cost-effective solution for large-scale, real-time deformation monitoring systems.

## 1. Introduction

Mining-induced geological hazards, including open-pit slope instability, ground subsidence, and tailing dam deformation, represent critical threats to operational safety and surrounding infrastructure, often resulting in catastrophic economic losses and casualties [1,2,3]. Real-time, millimeter-level deformation monitoring is therefore imperative for early warning systems and adaptive safety management in modern mining operations. Global Navigation Satellite Systems (GNSS), particularly the BeiDou Navigation Satellite System (BDS) with its dense constellation configuration and rapid convergence capabilities, have emerged as indispensable tools for continuous, all-weather monitoring in challenging geomatics environments [4,5,6].

However, achieving high-precision GNSS positioning in complex mining environments remains fundamentally constrained by signal propagation anomalies. Pit slopes, headframes, haul trucks, and irregular topographic features create severe non-line-of-sight (NLOS) conditions and anisotropic multipath effects, wherein signals arrive at the receiver via diffraction or reflection rather than direct line-of-sight (LOS) propagation [7,8,9]. These error sources exhibit strong spatial correlation with local obstruction geometry, resulting in carrier-phase measurements that violate the standard assumption of elevation-dependent noise characteristics. Consequently, conventional Real-Time Kinematic (RTK) positioning suffers from degraded ambiguity resolution success rates and centimeter-to-decimeter-level positioning errors, severely limiting the efficacy of BDS for safety-critical deformation monitoring.

Existing approaches to mitigating NLOS and multipath effects involve inherent trade-offs between real-time performance, environmental adaptability, and implementation complexity. Hardware-based solutions, including choke ring antennas and dual-polarized receivers, effectively suppress distant multipaths but prove economically prohibitive and geometrically inadequate for large-scale mining deployments [10,11]. Software-based strategies encompass stochastic model refinement, sidereal filtering (SF), and observation-domain signal decomposition. Stochastic models utilizing signal-to-noise ratio (SNR) or elevation-angle weighting assume azimuthal symmetry in observation quality—a condition fundamentally violated in mining environments with directional obstructions [12,13]. Function model-based techniques such as SF exploit orbital repeatability to correct multipaths yet require multi-day observation archives and are restricted to post-processing applications [14,15]. Similarly, coordinate-domain and observation-domain filtering methods (e.g., wavelet analysis and empirical mode decomposition) necessitate extensive historical datasets and substantial computational latency, rendering them unsuitable for real-time hazard warning systems [16,17,18]. Current methodologies fail to simultaneously satisfy three essential requirements for mining deformation monitoring: (1) real-time processing capability for immediate hazard detection; (2) anisotropic error modeling that accounts for direction-specific obstruction patterns; and (3) operational simplicity without dependency on multi-day satellite repeat arcs or expensive hardware modifications.

To bridge this gap, we present an adaptive RTK positioning approach of azimuth-integer-based elevation masking, which explicitly models site-specific obstruction geometry through discrete angular sampling. Unlike conventional elevation-cutoff or continuous azimuth-weighting strategies, the proposed method constructs a 360° elevation mask profile by quantizing the azimuth domain into integer-degree sectors and extracting minimum elevation angles for each sector. This discretization strategy preserves sharp transitions in obstruction boundaries—such as pit walls and infrastructure edges—while enabling real-time NLOS identification and adaptive stochastic weighting without multi-day data archives. Specifically, we introduce a virtual elevation-angle transformation that normalizes satellite geometry relative to the local mask profile, thereby down-weighting observations susceptible to diffraction and multipath while preserving high-integrity measurements.

The remainder of this paper is organized as follows. Section 2 details the double-difference (DD) RTK observation model and the theoretical foundation of the proposed masking framework. Section 3 describes the experimental validation conducted in a complex open-pit mining environment, followed by comparative performance analysis in Section 4.

## 2. Algorithm Adaptive RTK Positioning with Azimuth-Integer-Based Elevation Masking

In mining environments, RTK positioning performance is fundamentally constrained by non-line-of-sight (NLOS) signal propagation and anisotropic multipath effects induced by complex topography (e.g., pit walls, slope topography, trees, haul roads, and processing infrastructure). Conventional elevation-dependent stochastic models assume isotropic observation quality, thereby failing to discriminate between geometrically favorable low-elevation satellites and those subject to obstruction-induced signal degradation. To address this limitation, we propose an azimuth-integer-based elevation masking framework that explicitly models site-specific obstruction geometry through discrete angular sampling, enabling adaptive down-weighting of compromised observations while preserving the integrity of high-quality measurements.

### 2.1. DD Measurement Model for Short Baseline

The fundamental observation equation for short-baseline RTK positioning (typically <10 km) is formulated based on the code and the carrier phase double-difference (DD) operator, which effectively eliminates satellite and receiver clock biases, as well as ionospheric and tropospheric delays to first-order approximation through spatial differencing. For a baseline between the reference receiver (r) and rover (b) tracking satellites (s and t), the linearized DD observation model is expressed as follows:

(1) $$V=\nabla \Delta \phi -\left(\nabla \Delta \rho +\lambda \nabla \Delta N+\nabla \Delta M+\nabla \Delta \varepsilon \right)$$

where ∇Δ(⋅) denotes the double-differencing operator between receivers and satellites; ∇Δϕ represents the DD carrier-phase observations (converted to meters); ∇Δρ denotes the DD geometric range derived from a priori receiver coordinates; λ is the carrier wavelength; $\nabla \Delta N\in Z^{n}$ represents the integer ambiguity vector; ∇ΔM and ∇Δε account for multipath errors and measurement noise, respectively; and V denotes the residual vector.

Under the assumption of independent, zero-mean observation errors, the variance–covariance matrix (Q) of DD observations is initially modeled using an elevation-dependent weighting scheme:

(2) $$\begin{array}{c} \sigma_{\nabla \Delta }^{2}=a^{2}+\frac{b^{2}}{{sin}^{2}(\theta_{\mathrm{az}})} \end{array}$$

where $\theta_{\mathrm{az}}$ denotes the satellite elevation angle at azimuth (az), and the coefficients a and b represent the nominal noise level and elevation-dependent amplification factor, respectively. However, Equation (2) implicitly assumes azimuthal symmetry in signal quality, which is a condition violated in obstructed mining environments.

The estimation procedure employs a two-stage approach:

(1) Weighted Least-Squares (WLS) adjustment yields the float ambiguity solution ($\hat{N}\in R^{n}$) and its associated variance–covariance matrix ($Q_{\hat{N}}$);
(2) The LAMBDA (Least-squares AMBiguity Decorrelation Adjustment) method is applied for integer least-squares search and variance–covariance decorrelation [19,20]. The integer solution is subsequently validated using the Ratio Test, defined as the quotient of the squared weighted norms of the second-best and best integer candidates:
  (3) $$\begin{array}{c} \mathrm{Ratio}=\frac{{\left({\hat{N}}_{2}-\hat{N}\right)}^{T}Q_{\hat{N}}^{-1}\left({\hat{N}}_{2}-\hat{N}\right)}{{\left({\hat{N}}_{1}-\hat{N}\right)}^{T}Q_{\hat{N}}^{-1}\left({\hat{N}}_{1}-\hat{N}\right)}>R_{\mathrm{thres}} \end{array}$$
  where ${\hat{N}}_{1}$ and ${\hat{N}}_{2}$ denote the optimal and sub-optimal integer vectors, respectively. Following established conventions [21], the validation threshold is set to $R_{\mathrm{thres}}=3.0$ to ensure a judicious balance between solution availability and the ambiguity resolution success rate. Only upon satisfying Equation (3) are the fixed ambiguities (${\hat{N}}_{1}$) utilized to compute the final high-precision baseline solution; otherwise, the float solution is retained with correspondingly degraded precision.

### 2.2. Modeling of Elevation Mask Angle with Rounded Azimuth

To characterize the anisotropic observation environment prevalent in mining areas, we propose an azimuth-integer-based elevation mask model that discretizes the horizontal plane into integer angular sectors, enabling precise representation of irregular obstruction patterns (e.g., pit walls, machinery, buildings) that cannot be adequately described by continuous azimuth-dependent functions or conventional elevation cutoff angles. The modeling methodology comprises three sequential stages:

**Step 1**: LOS Data Extraction and Quality Control: Utilizing approximate receiver coordinates derived from standard point positioning (SPP) and 24 h broadcast ephemerides, line-of-sight (LOS) vectors for all visible satellites are computed at each epoch. To ensure arc continuity and mitigate cycle-slip contamination in the mask derivation, satellite arcs exhibiting observation gaps exceeding 5 min are systematically excluded from the modeling dataset.

**Step 2**: Azimuthal Discretization and Profile Generation: Continuous azimuth measurements, $\mathrm{az}\in [0^{\circ },360^{\circ })$, are quantized to the nearest integer degree, i∈{0,1,…,359}. For each discrete azimuth sector (i), the minimum elevation angle observed during the modeling period is extracted as the representative mask elevation, effectively capturing the worst-case obstruction geometry for that angular direction:

(4) $$\begin{array}{c} E_{\mathrm{mask}}=\left\{e_{i}|0\leq i<360\right\} \end{array}\begin{array}{c} e_{i}=min\left\{\theta_{\mathrm{az}}|i-0.5\leq \mathrm{az}<i+0.5\right\} \end{array}$$

where $e_{i}$ represents the elevation mask for the azimuth sector (i), and $\theta_{\mathrm{az}}$ denotes the original elevation angle from the LOS data. This discrete sampling strategy contrasts with conventional azimuth-dependent weighting approaches by preserving sharp transitions in obstruction boundaries critical for NLOS identification.

**Step 3:** Smoothing and Interpolation: To suppress outliers while preserving genuine topographic features, a moving average filter with an empirical window size of w=5 is applied to the extracted profile ($E_{\mathrm{mask}}$). Missing values for azimuth sectors (j) lacking direct observations are linearly interpolated between adjacent valid sectors (m and n) (m<j<n):

(5) $$\begin{array}{c} e_{j}=e_{m}+\frac{j-m}{n-m}\left(e_{n}-e_{m}\right) \end{array}$$

The resulting elevation mask model ($E_{\mathrm{mask}}$) provides a complete $360^{\circ }$ representation of the local obstruction environment without requiring satellite repeat-arc matching across multiple days, thereby enhancing computational efficiency for near real-time monitoring applications.

For azimuths without observations near the boundary, a minimum-elevation constraint was imposed. Specifically, linear interpolation was performed between azimuth 0° (or 360°) and the boundary scatter points. The interpolation formula has been added to the revised manuscript. Given the LOS points ($i_{0}$, $e_{i_{0}}$) and ($i_{1}$, $e_{i_{1}}$), find $e_{i}$ corresponding to any intermediate point (i). The formula is as follows:

(6) $$e_{i}=e_{i_{0}}+\frac{e_{i_{1}}-e_{i_{0}}}{i_{1}-i_{0}}({i-i}_{0})$$

### 2.3. Adaptive Integration of Mask Model into Stochastic Weighting

The elevation mask model serves dual functions: (1) explicit identification and exclusion of NLOS satellites ($\theta_{\mathrm{az}}<e_{\mathrm{az}}$) and (2) adaptive refinement of the stochastic model to down-weight observations susceptible to diffraction and multipath near the obstruction boundary. This integration addresses the critical limitation of conventional elevation-only weighting, which inadequately represents signal quality when low-elevation satellites correspond to diffracted or reflected paths rather than direct LOS. Therefore, we introduce a virtual elevation angle ($\theta_{{\mathrm{az}}^{\prime }}$) that normalizes the physical elevation ($\theta_{\mathrm{az}}$) relative to the local obstruction profile ($e_{\mathrm{az}}$,), effectively mapping the angular separation between the satellite and the nearest obstruction to a standardized elevation metric:

(7) $$\begin{array}{c} \theta_{{\mathrm{az}}^{\prime }}=\left\{\begin{array}{cc} \frac{\theta_{\mathrm{az}}-e_{\mathrm{az}}}{90^{\circ }-e_{\mathrm{az}}}\cdot 90^{\circ }, & \theta_{\mathrm{az}}<60^{\circ }\\ \theta_{\mathrm{az}}, & \theta_{\mathrm{az}}\geq 60^{\circ } \end{array}\right. \end{array}$$

where $e_{\mathrm{az}}$ is the interpolated mask elevation at the satellite’s azimuth, computed as follows:

(8) $$\begin{array}{c} e_{\mathrm{az}}=e_{i}+\left(e_{i+1}-e_{i}\right)\cdot \left(\mathrm{az}-\lfloor \mathrm{az}\rfloor \right),\;i=\lfloor \mathrm{az}\rfloor \end{array}$$

The $60^{\circ }$ threshold in Equation (6) reflects the empirical observation that signals above this elevation are generally immune to ground multipath and diffraction in open-pit mining configurations, whereas lower-elevation observations are progressively degraded by obstruction-induced signal attenuation. By selectively applying the down-weighting mechanism only to $\theta_{\mathrm{az}}<60^{\circ }$, the proposed strategy preserves the full weight of high-integrity measurements while penalizing geometrically vulnerable observations.

Substituting $\theta_{{\mathrm{az}}^{\prime }}$ for $\theta_{\mathrm{az}}$ in Equation (2), the refined variance model becomes the following:

(9) $$\begin{array}{c} \sigma_{\nabla \Delta \phi }^{\prime 2}=a^{2}+\frac{b^{2}}{{sin}^{2}(\theta_{{\mathrm{az}}^{\prime }}\cdot \pi /180)} \end{array}$$

This adaptive weighting strategy prevents the undue amplification of residuals that often precedes ambiguity fixing failures, thereby enhancing the ambiguity resolution success rate and overall reliability of RTK positioning in challenging mining environments. The integration effectively distinguishes between geometrically favorable low-elevation satellites and those subject to NLOS reception—a discrimination impossible with conventional isotropic stochastic models.

## 3. Experiment and Results

### 3.1. Experimental Arrangement and Data

**Figure 1:**
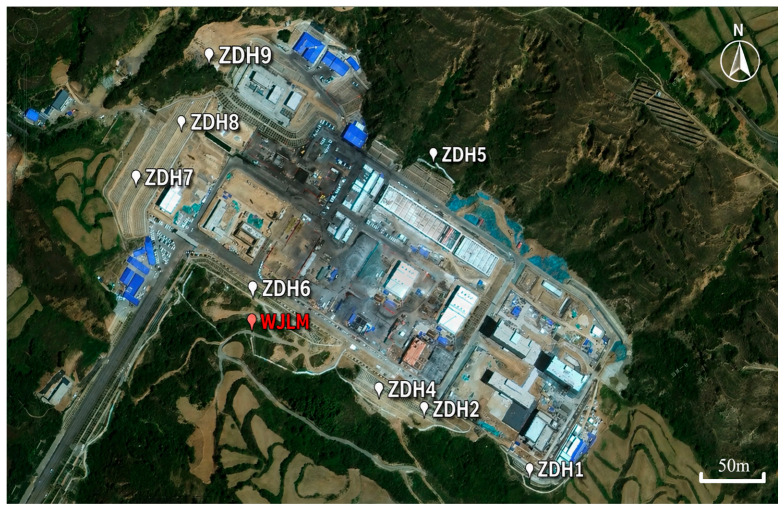
Illustration of the distribution map of the reference station (Red) and monitoring station (White).

To evaluate the performance of the proposed adaptive RTK positioning approach with azimuth-integer-based elevation masking under challenging operational conditions, BDS data over a 54 h period in 2025 from a deformation monitoring network with 9 stations was collected within the Wangjialing Mine of the China Coal Huajin Group Co., Ltd., which is located in Yuncheng, Shanxi province, China. Due to communication bandwidth constraints and the slow deformation nature of the region, the GNSS receivers were set to a 30 s sampling interval. There are eight monitoring stations (designated ZDH1–ZDH9, excluding ZDH3), which represent varying degrees of signal obstruction typical of industrial mining environments. WJLM was selected as the reference station because its distance from the active mining zone effectively eliminates mining-induced deformation, while its unobstructed sky visibility ensures the continuity and quality of reference data. Figure 1 illustrates the distribution map of the reference station (Red) and monitoring site (White). And Table 1 presents the baseline distance between the reference station (WJLM) and the eight monitoring sites. The baseline lengths range from 42.1 m (ZDH6) to 417.8 m (ZDH1), with all stations situated within a 0.5 km radius of the base site. The height differences between stations vary substantially, ranging from −0.8 m (ZDH4) to +36.9 m (ZDH8).

**Figure 2:**
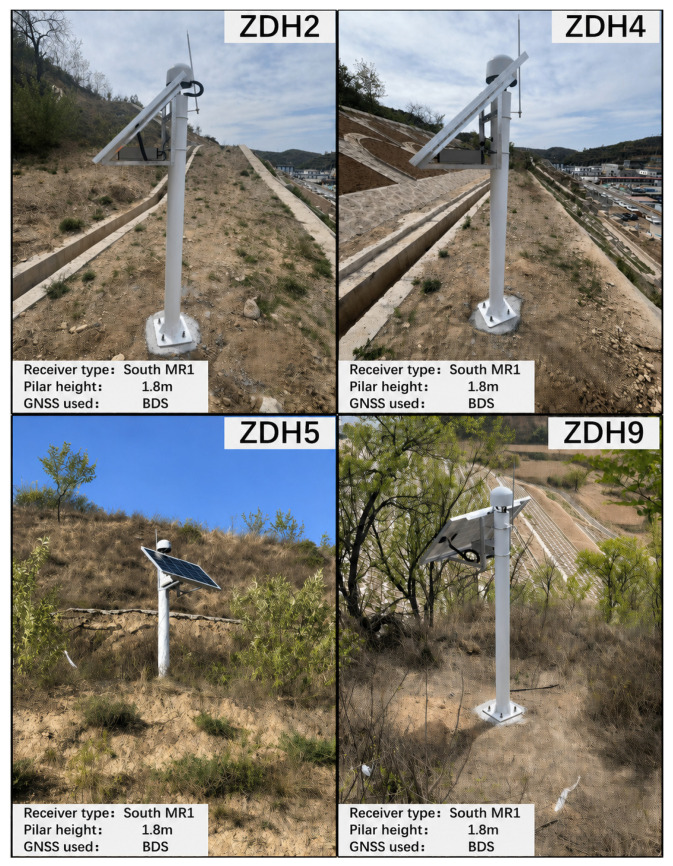
Typical measurement environment with four stations.

Figure 2 illustrates the challenging electromagnetic environment of representative rover stations deployed within the active mining complex. Subject to severe azimuthal constraints (elevation masks > 25°) imposed by surrounding industrial structures and haul infrastructure, these sites exhibit severe non-line-of-sight (NLOS) conditions and directional multipath contamination, thereby validating the necessity of azimuth-specific obstruction modeling.

### 3.2. Elevation Mask Angle Modeling

To mitigate non-line-of-sight (NLOS) signal interference, an azimuth-elevation mask was constructed for each GNSS receiver using the methodology outlined in Section 2.2. The procedure commenced with single-point positioning based on pseudo-orange measurements to obtain approximate coordinates for all monitoring stations. Subsequently, azimuth and elevation angles of all visible satellites were computed throughout the observation period using these station coordinates and broadcast ephemerides. Finally, station-specific azimuth-elevation masks were derived from these angular observations via Equation (3).

**Figure 3:**
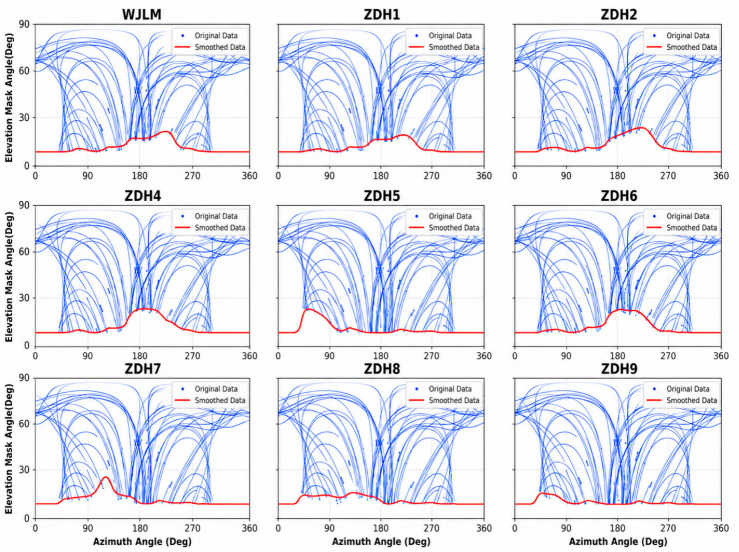
Elevation mask angle model of 9 stations during entire experimental period. Blue dots represent the cutoff elevation angles corresponding to integer azimuth angles, while the red solid line denotes the mask data smoothed using a moving window.

Figure 3 presents the elevation mask angle models of all sites. The azimuth-dependent elevation masks exhibit substantial spatial heterogeneity across the nine stations (Figure 3), primarily governed by local topographic configurations and anthropogenic mining structures. At the reference station (WJLM), the elevation cutoff angles demonstrate moderate azimuthal variation, ranging between approximately 10° and 20° (Figure 3), indicating relatively unobstructed sky visibility in the mining environment. Conversely, the typical monitoring station ZDH2 reveals pronounced directional asymmetry: the mask angles remain consistently low (~10°) within the 0–160° azimuth sector but escalate progressively from 10° to 25° between 160 and 360°. This azimuth-dependent undulation is attributable to the combined shielding effects of local terrain undulations and adjacent mining infrastructures, which selectively obstruct satellite line-of-sight paths at specific approach angles. Consequently, satellites with elevation angles below 25° become partially or fully occluded within the affected azimuth sectors, effectively reducing the observable constellation size at ZDH2 and potentially degrading positioning accuracy. It also can be seen that stations ZDH2 and ZDH4 have similar obstruction conditions, mainly concentrated within the azimuth range of 180–270°. The obstructions at station ZDH5 are mainly concentrated within 45–90°. ZDH9 also has partial obstruction within 45–90°, and there is significant vegetation obstruction near the station. Comparable azimuthal dependencies were observed across the remaining monitoring stations, suggesting systematic NLOS susceptibility in the mining area.

### 3.3. Results and Analysis

To rigorously validate the azimuth-dependent elevation mask model, a comparative analysis was conducted across three critical performance dimensions: satellite geometry strength and ambiguity resolution reliability via ratio statistics. Implementation of the mask model engendered a targeted reduction in satellite participation, decreasing the mean number of contributing satellites from 15.66 to 14.39 (an 8.1% reduction). This decrement reflects the algorithm’s capacity to discriminate between line-of-sight (LOS) and NLOS-contaminated signals, selectively excising observationally compromised satellites while preserving favorable geometric configurations. The model thus optimizes the quality–quantity trade-off inherent to constrained GNSS environments.

**Figure 4:**
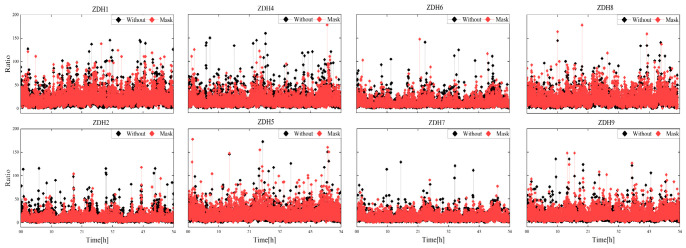
Comparison of the ratio values during entire experimental period. The black line represents the sequence without the elevation mask model, and the red line represents the sequence with the mask.

The ratio test sequences (Figure 4) reveal distinct temporal signatures between processing strategies. The unmasked solution (black trace) exhibits pronounced episodic depressions, with ratio values frequently oscillating near or below the empirical reliability threshold of 3.0, particularly during periods of high satellite turnover or low elevation geometry. Conversely, the masked solution (red trace) maintains consistently elevated ratio statistics with markedly attenuated fluctuation amplitudes, indicating enhanced phase observation consistency and reduced multipath-induced ambiguity degradation. The temporal stability of the red curve suggests robust NLOS mitigation across varying satellite constellations throughout the diurnal cycle. Table 2 give the comparison of the average ratio value of all stations during entire experimental period. Quantitative assessment reveals a statistically significant improvement in mean ratio values from 9.43 to 11.27 (a 19.5% relative increase). However, spatial heterogeneity characterizes the station responses (Table 1). Stations ZDH1, ZDH5, and ZDH8 exhibited substantial improvements (12.2%, 22.4%, and 20.7%, respectively), whereas ZDH2 demonstrated marginal change (9.6 to 9.8), suggesting pre-existing favorable observation conditions or limited NLOS contamination at this specific site. This differential performance underscores the site-specific efficacy of topographic masking, with the greatest benefits manifested at stations subject to severe azimuthal obstructions. The elevated ratio values signify reduced residual multipath contamination in the double-differenced phase observations, enabling more reliable integer least-squares estimation and minimizing the risk of Type I errors in ambiguity acceptance. 

**Figure 5:**
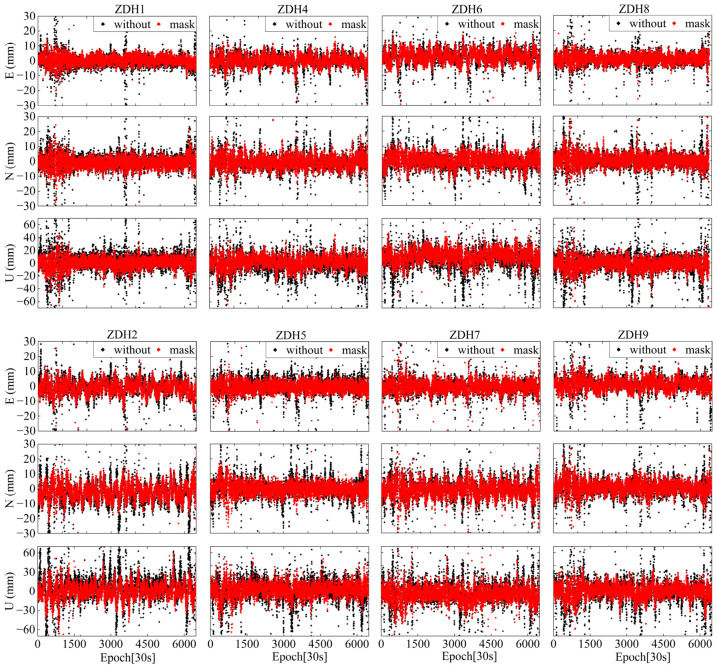
Comparison of RTK results of baselines in the east, north, and up (ENU) directions with and without the elevation mask model. Black dots denote results without the mask model, and red dots represent results with the mask applied.

Figure 5 presents a comparison of RTK results of baselines in the east, north, and up (ENU) directions with and without the elevation mask model. The reference values used to calculate the ENU components are the mean values of the fixed solutions of the baseline vectors. One can notice from the results that the elevation mask model can significantly lower the noise level and the outliers compared to the traditional solutions at eight baselines. The stability of the solution sequence with the elevation mask model is clearly better compared to the traditional solutions. Table 3 gives the average RMS of RTK results for baselines in ENU directions with and without the elevation mask model. To accurately obtain statistical results, abnormal values outside the range of ±30 mm in the horizontal direction and ±60 mm were not included in the statistics.

Component-specific statistics reveal differential improvement patterns across spatial dimensions. The vertical component (U) exhibits the most substantial gains, with RMS values decreasing from 15.2–50.7 mm to 8.4–14.8 mm across stations (average improvement: 64.3%). This pronounced vertical enhancement aligns with the geometric sensitivity of GNSS height determination to low-elevation satellite exclusion. Horizontal components (E/N) demonstrate commensurate though less dramatic improvements, with easting RMS reduced by 34.8–65.2% and northing RMS by 28.7–77.0%.

Station-specific analysis identifies ZDH2 and ZDH6 as exhibiting the most dramatic precision gains, particularly in the vertical component (ZDH2: 32.3 vs. 14.0 mm; ZDH6: 50.7 vs. 14.8 mm), suggesting severe NLOS contamination at these sites prior to masking intervention. In contrast, ZDH1 and ZDH8—characterized by moderate baseline RMS values—achieve more modest yet statistically significant improvements, indicating inherent environmental advantages or less obstructed sky visibility.

**Figure 6:**
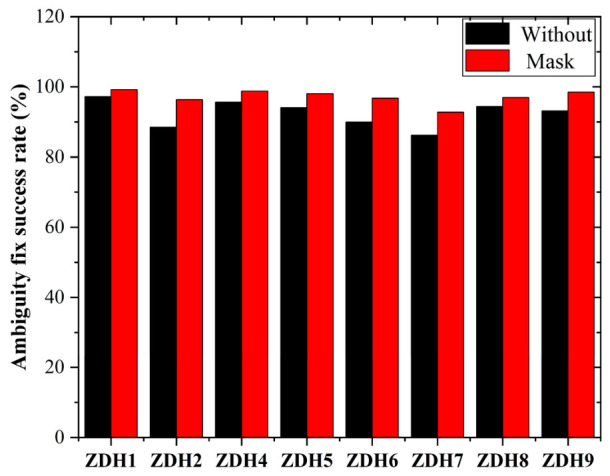
Comparison of ambiguity fix success rate of RTK results for eight baselines.

Figure 6 and Table 4 show that the performance of the azimuth-dependent elevation mask model in facilitating reliable integer ambiguity resolution was quantified through comparative analysis of fix success rates across the eight-baseline network. The masked solutions demonstrate systematically elevated success rates relative to conventional processing, achieving a network-wide average of 97.2% compared to 92.4% for unmasked solutions—corresponding to a statistically significant improvement of 4.8%. This uniform elevation of success rates above the critical 95% threshold for reliable RTK operation corroborates the ratio test improvements reported previously. By excising NLOS-contaminated observations that introduce ambiguity search space degradation, the mask model ensures that only high-integrity phase measurements contribute to the integer least-squares estimation, thereby minimizing the risk of incorrect ambiguity acceptance (Type I errors) across the diurnal observation cycle.

## 4. Conclusions and Discussion

This study was designed to address the limitations of conventional elevation-dependent stochastic models in open-pit mine deformation monitoring, where NLOS signals and anisotropic multipath frequently degrade RTK ambiguity resolution and positioning accuracy. To achieve this objective, an adaptive RTK positioning framework based on an azimuth-integer-based elevation mask was developed. By discretizing the horizontal plane into 360 azimuth sectors, extracting the minimum elevation angle for each sector from 24 h LOS observations, and introducing a virtual elevation-angle transformation, the proposed method enables site-specific and direction-dependent down-weighting of diffraction-susceptible observations without additional hardware or empirical threshold tuning.

The experimental results obtained from 54 h of BDS observations at eight monitoring stations in the Wangjialing open-pit mine confirm that the proposed strategy is consistent with the study objective and effectively improves deformation monitoring performance in obstructed environments. Although the average number of participating satellites decreased by 8.1% (from 15.66 to 14.39), the observation quality was significantly improved. The ambiguity validation ratio increased by 19.5% (from 9.43 to 11.27), and the fix success rate increased from 92.4% to 97.2%, exceeding the 95% reliability threshold at all stations. In addition, the RMS errors in the east, north, and up components were reduced by 34.8–65.2%, 28.7–77.0%, and 44.8–70.8%, respectively. These results demonstrate that explicitly modeling azimuth-dependent obstruction geometry can effectively suppress NLOS-related errors and provide a practical, cost-effective solution for robust high-precision deformation monitoring in complex mining areas.

Nevertheless, several limitations should be acknowledged. The current experiment focused on static monitoring stations, so the performance of the method in kinematic applications remains uncertain. At the same time, the mask model was derived from 24 h LOS data and assumes that the local obstruction pattern remains relatively stable. In environments with rapidly changing equipment layouts or temporary obstructions, the mask may need frequent updating. Future work will therefore focus on multi-site validation, extensions to multi-constellation and kinematic scenarios, and the development of adaptive real-time mask-updating strategies under changing observation conditions.

## Figures and Tables

**Table 1:** Difference between baseline length and altitude.

Monitoring Site	Base Site	Length of Baseline (Unit: m)	Height Difference (Unit: m)
ZDH1	WJLM	417.8	+2.4
ZDH2	WJLM	238.6	+6.6
ZDH4	WJLM	188.7	−0.8
ZDH5	WJLM	305.1	+9.4
ZDH6	WJLM	42.1	+9.55
ZDH7	WJLM	248.3	+36.2
ZDH8	WJLM	280.6	+36.9
ZDH9	WJLM	333.7	+12.3

**Table 2:** Comparison of the average ratio value of all stations during entire experimental period.

Station	ZDH1	ZDH2	ZDH4	ZDH5	ZDH6	ZDH7	ZDH8	ZDH9
without	18.9	9.8	15.7	14.7	10.8	9.6	16.9	13
mask	21.2	9.6	15.4	18.0	11.1	10.4	20.4	15.8

**Table 3:** Comparison of the average RMS of RTK results for baselines in ENU directions with and without the elevation mask model (the measurement unit is mm).

Station	ZDH1		ZDH2		ZDH4		ZDH5	
	Without	Mask	Without	Mask	Without	Mask	Without	Mask
E	4.3	2.8	13.5	4.7	8.4	3.4	8.0	3.1
N	5.2	3.7	8.9	5.7	21.5	5.2	17.1	3.9
U	15.2	8.4	32.3	14.0	39.8	10.5	42.3	10.8
**Station**	**ZDH6**		**ZDH7**		**ZDH8**		**ZDH9**	
E	14.4	4.8	16.1	6.1	4.9	2.9	12.3	3.7
N	15.7	3.9	23.7	6.3	6.3	4.5	20.6	4.8
U	50.7	14.8	36.8	13.6	15.7	8.9	42.8	14.2

**Table 4:** Comparison of ambiguity fix success rate of RTK.

Baseline	Without	Mask	Baseline	Without	Mask
ZDH1	97.22	99.21	ZDH6	89.96	96.77
ZDH2	88.54	96.34	ZDH7	86.25	92.77
ZDH4	95.64	98.79	ZDH8	94.39	96.91
ZDH5	94.08	98.05	ZDH9	93.17	98.50

## Data Availability

The datasets analyzed in this study are managed by the China Coal Xi’an Design Engineering Co., Ltd., which can be available on request from the corresponding author.
